# Involving children and adolescents with type 1 diabetes in health care: a qualitative study of the use of patient-reported outcomes

**DOI:** 10.1186/s41687-023-00564-0

**Published:** 2023-03-02

**Authors:** Rikke Bjerre Lassen, Caroline Bruun Abild, Kurt Kristensen, Lene Juel Kristensen, Jens Thusgård Hørlück, Annesofie Lunde Jensen

**Affiliations:** 1 Steno Diabetes Center Aarhus, Aarhus University Hospital, Aarhus, Denmark; 2 Department of Clinical Medicine, Aarhus University, Aarhus, Denmark; 3 DEFACTUM, Aarhus, Denmark

**Keywords:** Qualitative study, Pediatrics, Diabetes, Patient-reported outcomes, DEPS-R, WHO-5, Patient involvement, Applied research

## Abstract

**Background:**

Within pediatric health care services, Patient-reported Outcomes (PROs) regarding the patient’s health status are mainly used for research purposes in a chronic care setting. However, PROs are also applied in clinical settings in the routine care of children and adolescents with chronic health conditions. PROs have the potential to involve patients because they ‘place the patient at the center’ of his or her treatment. The investigation of *how* PROs are used in the treatment of children and adolescents and how this use can influence the involvement of these patients is still limited. The aim of this study was to investigate how children and adolescents with type 1 diabetes (T1D) experience the use of PROs in their treatment with a focus on the experience of involvement.

**Results:**

Employing Interpretive Description, 20 semi-structured interviews were conducted with children and adolescents with T1D. The analysis revealed four themes related to the use of PROs: Making room for conversation, Applying PROs under the right circumstances, Questionnaire structure and content, and Becoming partners in health care.

**Conclusions:**

The results clarify that, to some extent, PROs fulfill the potential they promise, including patient-centered communication, detection of unrecognized problems, a strengthened patient-clinician (and parent-clinician) partnership, and increased patient self-reflection. However, adjustments and improvements are needed if the potential of PROs is to be fully achieved in the treatment of children and adolescents.

## Background

The development and implementation of Patient-reported Outcomes (PROs) within pediatric health services is rapidly increasing in today’s health care system, particularly in the treatment of children and adolescents with chronic conditions, such as type 1 diabetes (T1D) [1]. A systematic review of the literature evaluating the impact of the use of PROs as an intervention in routine clinical care for such patients has found that integration of PROs increase health-related quality of life, clinical outcomes, and quality of care, underlining the legitimacy of applying PROs in pediatric care settings [2]. PROs are directly reported by the patient through questionnaires without interpretation of the patient's response [3]. PROs is an 'umbrella term', involving both reports from patients about their health status associated with the health care they recieve, such as health-related quality of life, and reports from patients about their experiences with health services, e.g., treatment satisfaction [4].

PROs enable early detection of symptoms and prevention of disease complications [5]. PROs may qualify patient-clinician communication on topics such as health-related quality of life by involving the patient’s point of view in care [6]. PROs may also help the patient gain a better understanding of his or her disease, which can promote self-management [7].

An extensive burden of self-management tasks is placed on children and adolescents with T1D (and their parents), including counting carbohydrates, planning physical activity, checking blood glucose levels, and administering insulin with the goal of maintaining glycemic control [8–10]. Furthermore, adolescence represents a time of transition characterized by growing independence and decreasing parental responsibility [11]. Consequently, children and adolescents with T1D have considerable knowledge about their own health [12–14]. Therefore, their perspectives on disease, treatment, and well-being are recognized as a means to improve treatment (e.g., patient-clinician communication, patient satisfaction, and patient compliance) [13–15]. The perspectives of children and adolescents can be included by using PROs. The application of PROs represents an increasing interest in the concept of patient involvement, defined as the processes of care, in which the individual patient's preferences, resources, and life situation are taken into consideration [16, 17]. Enhancing patient involvement can improve both services and health outcomes, underlining the importance of a patient-centered approach to care [18, 19].

Research has primarily focused on the development and validation of PROs and on the use of PROs to assess the impact of treatment on e.g., symptom burden, physical functioning and health-related quality of life among children and adolescents with T1D [5, 20–22]. Conversely, little attention has been given to the perspectives of children and adolescents on the use of PROs in the clinical care setting. A qualitative study has shown that pediatric patients consider PROs to promote clinicians’ focus on the illness, narrow access to care, and cause uncertainty about what is safe to reveal to clinicians [23]. Patients have noted concern about how their individual experiences are represented within the fixed structures of PROs [23]. Moreover, studies have found that pediatric patients raise concerns about the degree of literacy needed to understand and answer abstract or vague questions [24, 25]. Finally, knowledge is limited on how these patients experience being involved, especially when they enter adolescence and gradually become more responsible for their care [26, 27]. One study of how adolescents with T1D experience the partnership with clinicians in the transition from pediatric to adult services found that the adolescents did not have the perception of being part of a dialogue, and that the transition process was not based on maturity and individual needs and wishes, even though this was the intention [17].

These studies emphasize the importance of focusing on *how* PROs are used rather than on the PROs themselves. Reviewing the studies also points out a gap in the existing literature, investigation of the patient-involving opportunities of PROs in the treatment of pediatric patients. Therefore, the aim of the present study was to investigate how children and adolescents with T1D experience the use of PROs in their treatment with a focus on the experience of involvement. This aim was pursued in order to expand our knowledge on how and when PROs are experienced as useful to children and adolescents with T1D and to optimize the use of PROs in their treatment.

## Methods

### Study design

To investigate how children and adolescents with T1D experience the use of PROs, this study was guided by the qualitative methodology Interpretive Description (ID) and comprised semi-structured interviews. The purpose of using ID was to answer the research question and provide clinical practice with a research-based choice of action [28].

### Setting

The study was conducted in the pediatric outpatient clinic at Steno Diabetes Center Aarhus, Aarhus University Hospital, in Central Denmark Region. Children and adolescents with T1D attend the outpatient clinic four times a year. Three appointments are ‘basic’ consultations at which well-being and glucose levels are discussed, among other things. The fourth appointment is an extended consultation, which consists of a number of examinations, including blood pressure, blood, and urine tests. Consultations involve communication with clinicians, in this case a doctor, dietician, and/or nurse, about diabetes management.

**Fig. 1:**
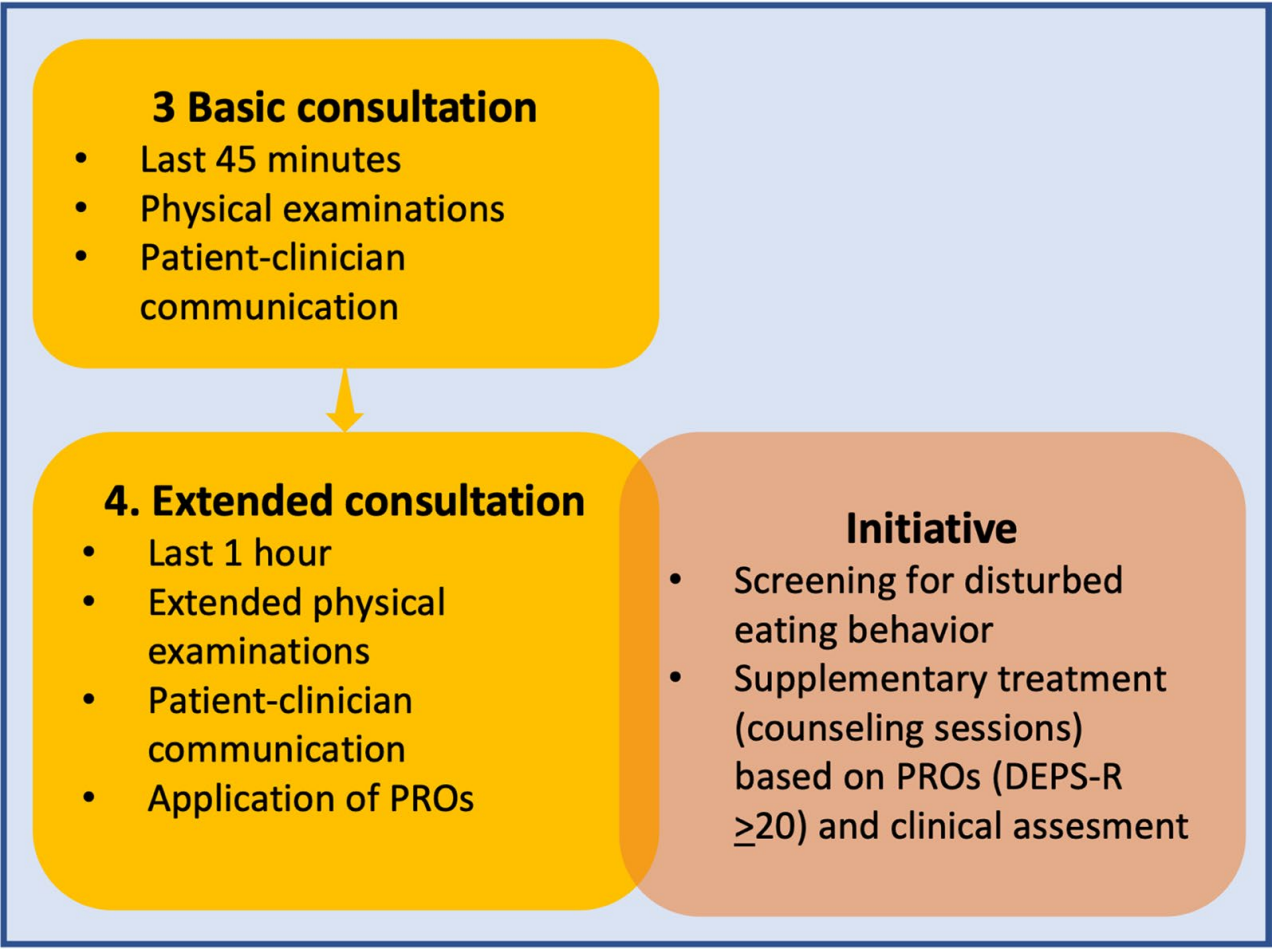
Annual process of standard treatment and the initiative in the outpatient clinic

An initiative was started at Steno Diabetes Center Aarhus in July 2018 to screen for disturbed eating behavior (e.g., skipping meals and feelings of shame associated with eating) among children and adolescents with T1D in order to prevent eating disorders and disease-related complications. As part of the initiative, all children and adolescents aged 11 to 18 years were asked to complete a questionnaire seven days prior to their extended consultation either at home, or if not completed at home, in the waiting room at the outpatient clinic. PROs from the questionnaire were intended as a tool to support patient-centered communication and identify symptoms and problems. The PROs applied were the Diabetes Eating Problem Survey-Revised (DEPS-R) (containing 16 questions designed to assess disturbed eating behavior specific to T1D, including insulin restriction to lose weight) [20], the generic WHO-5 Well-Being Index (consisting of five questions designed to capture emotional well-being) [21], five questions on involvement (concerning patient-clinician communication), one question about the patient’s overall satisfaction with the latest consultation in the clinic, and an additional comments area. These PROs were gathered into one questionnaire in the web-based PRO system Ambuflex [29]. Responses were recorded and scored on a scale with six categories of answers (Appendix I), and PROs were presented to clinicians using the usual electronic medical record system. Higher DEPS-R scores indicated greater disturbed eating. Based on empiricism, a pre-determined cut-off score for disturbed eating was ≥ 20, which indicated patients with a level of disturbed eating warranting supplementary treatment (Fig. 1) [30]. Clinicians were trained to interpret questionnaire answers and to discuss these answers with the patient.

On the basis of PROs, particularly the DEPS-R score, and a clinical assessment, patients could be offered supplementary multidisciplinary treatment by a psychologist, psychiatrist, nurse, pediatrician, and/or a dietician. Figure 1 outlines the process of standard treatment and the initiative in the clinic.

### Sampling

Children and adolescents with T1D were invited to participate in individual interviews. They were sampled purposively; participation criteria were patients with T1D (or similar diabetes type) aged 11 to 18 years who completed the questionnaire at least two times [28]. 11 was chosen as a lower age boundary due to the fact that children in this age have the appropriate cognitive skills to read and understand questions and to select answers matching their perspectives [13]. Furthermore, both the DEPS-R and the WHO-5 has been validated in T1D patients in this age group [20, 21]. With the purpose of obtaining nuanced data, patient age and gender were kept in mind during recruitment.

Participants were recruited in collaboration with clinicians in the clinic. Before the annual extended consultation, an information letter about the study was sent to patients meeting the participation criteria. Prior to the consultation, the clinicians were informed that patients had received the information letter. The clinicians were asked to invite patients to participate. Patients were invited if they had experiences with PROs and had an interest in spending time with a researcher to explain those experiences [28]. Patients also had the opportunity to contact the first author if they wanted to participate.

### Data collection

Data were collected from December 2020 to October 2021 in 20 semi-structured interviews. The first author (RBL) conducted the interviews, except for five interviews that were conducted by a co-author (CBA). These authors worked as qualitative researchers and were previously affiliated with the clinic (as a student assistant and a dietician). The intention of the interviews was to explore the participants’ experiences and reflections related to answering the questionnaire and the use of PROs in the consultation, including the patient-involving potential of PROs. A semi-structured interview guide was applied and adjusted concurrently with data collection and analysis in accordance with ID [28]. The interview guide had four themes: Practical aspects of completing the questionnaire (e.g., ‘Tell me about the last time you completed the questionnaire’), Content of the questionnaire (e.g., ‘Are any of the questions difficult to answer?’), Experiences assessing one’s own condition and needs (e.g., ‘How has it been to assess your attitude towards eating and diabetes treatment?’), and Use of PROs in the consultation (e.g., ‘Tell me how the questionnaire was used in the conversation with your doctor/nurse’). Select questions from the questionnaire were read to participants to support conversation during the interview. The interviews took place in the clinic or were carried out using telephone or video communication. Interviews were audio-recorded and had a duration of 11 to 48 min (mean 31 min).

### Data analysis

Interviews were transcribed verbatim by the first author, a secretary, or a student assistant. In line with ID, data analysis was performed by the first author inductively and concurrently with data collection and comprised three analytical phases: initial reading of the interview transcripts, de-contextualization of the data, and re-contextualization of the data. The analysis also contained ongoing memo writing, noting immediate meanings and relationships in the data. Interview transcripts were organized and coded in the qualitative data analysis software NVivo. First, the transcripts were read through to get an impression of the data. Second, open coding of data was carried out to assign headings/codes to text segments to identify themes and patterns. As the analysis proceeded, some codes were modified or eliminated either because they revolved around the same topic or because they were not relevant to the study objective [28]. Finally, the dimensions of participants’ experiences with PROs were gathered into four themes. In the presentation of findings, ‘ID’ followed by a number refers to a specific participant.

## Results

### Participants

A total of 20 children and adolescents aged 11 to 18 years (mean 14.85 years) with T1D participated in the study. Further characteristics are described in Table 1.

**Table 1:** Sample characteristics

Characteristic	Total sample (*N* = 20)
Age, years	14.85 [11–18]
*Gender*	
Male Female	9 (45) 11 (55)
*Diabetes type*	
T1D Secondary diabetes	18 (90) 2 (10)
Completed questionnaires	2.6 [2–5]

Values are given as mean [range] or n (%)

Findings from the analysis were categorized into four themes: Making room for conversation, Applying PROs under the right circumstances, Questionnaire structure and content, and Becoming partners in health care.

### Theme 1: making room for conversation

Overall, the use of PROs was described by the participants as promoting and improving conversation in their consultations with clinicians. However, participants identified a valuable topic of conversation, namely social health, that was not part of the PROs.

First, the DEPS-R questionnaire had a new focus on meals, body, and weight. Some participants found it rather difficultto answer questions about these, from their point of view, personal topics, thereby crossing personal boundaries:

“Sometimes it’s difficult to answer because I know that I have to answer honestly, but there are some things that I’m ashamed of…” (ID3)

This participant linked her own eating behavior with shame and struggled to answer questions about this topic area. PROs from DEPS-R facilitated topics of conversation other than standard treatment (e.g., blood glucose levels and insulin treatment), which promoted conversation from the participants' point of view. Talking about eating behavior was experienced as a delicate and vulnerable situation because the participants’ thoughts and feelings around the topic were exposed. Furthermore, the participants tended to concern about parental disapproval if such topics were identified. Even though the change in focus seemed intense to participants, they appreciated it. Diabetes management and meals were closely connected to each other by the participants and by introducing DEPS-R in the consultation the participants’ everyday lives became a bigger part of their care. Thus, PROs worked as a tool to identify and discuss otherwise neglected problems. Participants viewed this as an improvement in the patient-clinician dialogue.

The conversation focused on the individual by expressing issues or concerns that the patient reported, which had a positive impact on the participants’ views of PROs. One participant used the additional comments area to supplement the questionnaire with a topic of importance to her, namely the transition from the pediatric clinic to the adult clinic, which expanded conversation in the consultation:

“It was probably because I was turning 18. In that situation, the questionnaire helped me go into some of the things that you don’t usually talk about.” (ID5)

This quotation exemplifies the perception of PROs as an instrument to guide the conversation towards the participants’ specific situation, own values, and strategies in order to make patient-clinician communication patient-centered. Moreover, the quotation underlines the significance of using PROs in an < 18 population without parents being involved.

Conversely, no focus on the social dimensions of disease-related issues was present in the questionnaire, even though such a focus was cherished among participants. Most participants expressed an interest in incorporating questions about how their social environment (e.g., family, friends, school, or hobbies) interacts with their diabetes, and acknowledged the relevans of a dialogue about social health with clinicians. The content of such questions was exemplified by the following quote:

“It could be whether your diabetes sometimes prevent you from getting together with someone after school, or if your diabetes sometimes stops you from taking part in games.” (ID15)

The introduction of such questions was viewed by participants as a possibility for improving patient-clinician communication, allowing for dialogue about the complex interplay between disease and social environment.

### Theme 2: applying PROs under the right circumstances

Specific conditions under which the questionnaire was introduced and used proved pivotal to participants’ appreciation of the application of PROs in their treatment.

Most of the participants explicitly identified and decribed PROs as a tool supporting the clinicians’ work. According to the participants, PROs helped clinicians prepare for the consultation. Generally, participants experienced that PROs were discussed in the consultation as a result of the clinicians' preparation:

“It’s nice that they [the clinicians] say, ‘I can tell from your response that things aren’t well,’ and then you can talk about it.” (ID7)

When participants got feedback on their questionnaire response, it became clear to them how PROs were used in their treatment.

Participants reflected on the clinicians' communication about the questionnaire, and several of them expressed a lack of information about the aim of questions on involvement and the DEPS-R. Some participants felt that they appraised and criticized the clinicians' work by answering the questions on involvement:

"I hope that, by answering that I don't feel involved, my doctors won't be mad or sad." (ID4)

The quotation illustrates not only the discomfort that some participants experienced when answering the questions on involvement, but also a misconception about the purpose of the questions. Other participants were not aware that the DEPS-R is used as a screening tool for disturbed eating. This lack of awareness may be due to missing information about the entire questionnaire, and it underlines the significance of clearly communicating the aim of the applied PROs in the clinic.

Participants who did not understand the purpose of answering questions concerning meals, body, and weight (DEPS-R) found these questions irrelevant. They managed their diabetes according to what they ate and how they exercised. Therefore, items about overeating, weight control, and vomiting were questioned by these participants. A participant with secondary diabetes explained how these questions lacked relevance to her:

“I also have diabetes, but I think that some of the questions were aimed at those who only have diabetes and might have struggled a little more with being overweight… The questions were a little bit like ‘what do you want the most: to manage your disease or be skinny?’ And I have always struggled with gaining weight and been underweight my entire life. But I manage my disease, so I don’t quite understand the questions.” (ID6)

Conversely, participants who expressed knowledge of the aim of the DEPS-R (regardless of problems with disturbed eating) viewed the questions as relevant and important. According to these participants, such questions were able to call forth the proper care for the patients in need of it.

The relevance of the questions on involvement was also viewed differently among participants. Some saw these questions as unnecessary by virtue of already feeling involved in the treatment. Others appreciated being asked about their involvement as they felt the most qualified to answer questions about this topic.

### Theme 3: questionnaire structure and content

Participants reflected on issues concerning both the structure and content of the questionnaire.

The temporal frame in which questions were being asked troubled participants, such as when they were asked about patient-clinician communication in their last consultation approximately 3 months earlier. Health status changes between questionnaire completion a few days before the consultation and the in-clinic consultation made other participants view the PROs as invalid.

Participants problematized the standardized response categories. The most common concern was how the complexity of an individual life was represented in the secure categories:

“There weren’t any categories that made sense to me, so I just crossed out the lowest. I didn’t belong to any of the levels.” (ID2)

This quotation clarifies the importance of supporting PROs with dialogue to ensure that patients are not reduced to simple categories and numbers.

Specific formulations were viewed as difficult to understand. Distinguishing the five questions on involvement was challenging for most participants, and they suggested combining some of these questions without compromising the assessment of their involvement in care. Some participants found it difficult to understand what ‘involvement’ was, as the concept seemed too abstract to them. The wording ‘I try to eat to the point of spilling ketones in my urine’ was described as unclear, as ‘doctor language’, or as ‘adult language’. This led to potentially invalid answers. No age differences were found in difficulties understanding the questions.

### Theme 4: becoming partners in health care

The relationship between participants and clinicians was identified as significant to participants, and the PROs played an important part in supporting this relationship. For some participants, parents were involved in the treatment, and the completion of the questionnaire and the subsequent use of PROs in the consultation influenced the relationship between parents, patients, and clinicians. Ultimately, this led to increased patient (and parental) involvement. Furthermore, the application of PROs showed potential to promote a partnership between participants and clinicians through critical self-reflection.

Some participants did not involve their parents while filling in the questionnaire. They viewed it as an opportunity to share ‘secret’ information with clinicians, which resulted in a feeling of having a special bond with clinicians. One participant described the additional comments area as follows:

“It gives me the chance to say some things that I might not want to say in front of my parents… Because I wanted to keep some things between me and my nurse.” (ID3)

By letting the individual set the agenda through PROs, the consultation was personalized, and the participant and the clinician(s) became equal partners.

Participants who had difficulty understanding questions explained that their parents helped them complete the questionnaire. These parents took part in a PRO-based dialogue with their children and clinicians, indicating a partnership between parents, patient, and clinicians. Thus, the use of PROs led to a new way of involving both participants and parents in care, ultimately increasing patient and parent involvement.

Based on PROs, supplementary treatment, such as counseling sessions with a psychologist, was offered to specific participants. They took part in deciding on and planning the treatment in collaboration with clinicians. Consequently, PROs enhanced the participants’ experience of having ownership of their own treatment, which strengthened their autonomy and supported their partnership with clinicians.

A group of the participants found that completing the questionnaire helped them gain a better understanding of their disease. By reflecting on the questions presented in the questionnaire, the participants obtained new perspectives on their diabetes management:

“You get a little… Do people really do such things? Trying to eat in a way so that they can spill ketones. I’m thinking ‘shit, is it possible to get that far out?’” (ID11)

Another participant pointed out how specific questions had been eye-opening to her:

“Well, I have definitely faced some facts by answering them [the questions]. But it isn’t necessarily negative. For instance, this one, ‘When I overeat, I don’t take enough insulin to cover the food.’ When I answer that, I’m thinking ‘that’s actually pretty stupid. Why do you do that?’” (ID7)

Through self-reflection, participants were able to take a stand regarding their disease and care. By expressing such reflections to clinicians, children and adolescents with T1D may become more active partners in the patient-clinician collaboration. In addition, these reflections can increase the patients’ self-management. Yet, participants expressing an educational aspect of PROs were also the ones experiencing problems with disturbed eating. Other participants did not reflect on the particular questions; they filled in the questionnaire and put it aside. Moreover, the oldest participants were the ones reflecting on their self-management, which makes sense on account of the transitional stage of their treatment.

## Discussion

Overall, this qualitative study found that the participants experienced PROs as promoting and improving the conversation in the consultation by including new topics (exceeding standard treatment) and focusing on individual issues, thereby creating patient-centered communication and increased patient and parental involvement.

This study showed that PROs from the questionnaire did not reflect the social dimensions of the patient’s disease. This deficiency may be due to challenges regarding the implementation of new initiatives in established practices in the health care system. Questions in DEPS-R focused on physical health (e.g., weight and blood glucose levels), leaving consultations to be dominated by a biomedical agenda. However, study participants emphasized the importance of assessing the interaction between disease and social environment. These findings are consistent with studies showing that clinicians prioritize objective PROs (patient-reported physical health), whereas pediatric patients prioritize subjective PROs (patient-reported mental and social health) [31–34]. Furthermore, previous studies have found that children and adolescents with chronic conditions want to be met as individuals and acknowledged holistically [15, 17], stressing the significance of PROs in being able to evaluate other aspects of health than just the physical aspects.

In addition to the physical health-focused aspects of the questions in DEPS-R, the participants in this study characterized them as personal. Even though participants appreciated these new topics, they also experienced them as difficult to answer due to their personal nature, which may be due to the taboo around both male and female body ideals [35]. Clinicians should be aware of this when using PROs from the DEPS-R in the consultation. Participants valuing the change in focus are consistent with the aforementioned priority of subjective PROs. Moreover, the result is compatible with existing knowledge on PROs in the treatment of adult patients, including the detection and legitimization of unrecognized problems [7, 36, 37].

The approach of using PROs to identify patients’ individual needs and delivering patient-centered care can be questioned, as individual experiences are quantified through standardized questionnaires. On the one hand, participants in this study found the response categories to lack nuance and to be unable to measure the complexity of their lives. Similar perspectives were found in a study of pediatric patients’ views on PROs, leading the patients to suggest an unstructured, flexible questionnaire format. They suggested using pictures and symbols instead of numbers and words [23]. On the other hand, this study’s participants felt involved in their treatment in different ways as a result of applying PROs. Through the use of PROs, participants actively participated in a partnership with clinicians, and they became aware of their own self-management, which is comparable with studies of PROs in adult care [6, 7, 36, 38–42]. Notably, patients in the above-mentioned study of pediatric patients’ views on PROs considered PROs to be a method that promoted the clinicians’ focus on illness, thereby narrowing the focus on the patient-clinician relationship [23]. This contrasts with the strengthened relationship between participants and clinicians found in this study, clarifying that PROs should not be overarching in the conversation but only create a basis for dialogue [40]. Yet, the PROs used in the aforementioned study involved only health status reports in terms of symptoms and health-related quality of life, which may explain the differences in youth perspectives on PROs [23].

In some cases, lacking information about the aim of the questionnaire made study participants view the questionnaire as uncomfortable. The misconception of the questions on involvement being an evaluation of the clinicians led to a fear of criticizing them, even though the real purpose of these questions was to ensure patient-centered care. Thus, pediatric patients need a clear explanation about the purpose of using PROs in their treatment in order to avoid uncertainty about what is safe to reveal to clinicians.

The implementation of PROs in the treatment of pediatric patients should be considered carefully, as the use of PROs seems to entail some challenges for these patients. Even though this study found no association between low age and troubles completing the questionnaire, it seems reasonable to take patient age into account when deciding on applying PROs in pediatric health care. Yet, studies have shown that age should not be the only indicator of a pediatric patient’s ability to independently self-report. These studies have elucidated that literacy (reading and writing) skills determine the ability of children and adolescents to complete a questionnaire in a valid manner [24, 25, 43, 44]. Reading assistance from parents (as in this study) or clinicians based on pediatric patient choice should be a possibility to ensure data quality [25]. Conversely, some of the younger participants did not have any difficulties understanding the term ‘involvement’ and more technical terms. It is plausible that exposure to a treatment-intensive and chronic disease, such as T1D, provided an introduction to medical terms and health-related vocabulary that normally would be absent through childhood [25, 44].

This study has some limitations. The sample size was adequate for a qualitative study, and age and gender varied among participants, making the results relatively representative of children and adolescents with T1D. Yet, the sample size seemed too small for conclusive findings about subgroup differences, e.g., differences between patients with and without disturbed eating or differences between adolescents and younger children. In addition, data were collected by two authors, but only analyzed by one of these authors. To ensure a comprehensive understanding of the study subject, investigator triangulation was used as a strategy to test validity [45].

This study only focused on patients’ experiences with PROs and involvement. Other perspectives should be explored to investigate additional actions in relation to PROs. Thus, further research should involve other qualitative methods, samples of pediatric patients with other chronic conditions, and clinicians’ and parents' perceptions of PROs in pediatric health care.

## Conclusion

Applying PROs in the treatment of children and adolescents with T1D leads to an experience of focused consultation with new topics of conversation and a patient-centered approach, which were highly valued by participants in the present study. For participants to acknowledge the benefits of the PRO application, the aim of the entire questionnaire needs to be clear, and for PROs to be valid, questions have to be understandable. In some instances, the application of PROs can strengthen the patient-clinician and parent-clinician partnerships, which increases patient (and parental) involvement. However, the potential benefits of using PROs in this patient group will not necessarily be achieved unless the questionnaire structure and age and literacy skills of the patients are thoroughly considered. In addition, asking about the interaction between disease and social environment will allow for a holistic assessment of the patient’s health.

## Data Availability

The dataset generated and analyzed is not publicly available but is available from the corresponding author on reasonable request.

## Appendix I: Content and structure of the web-based AmbuFlex questionnaire

**Table:**

Total number of items	27
*WHO-5 Well-Being Index*	
Number of items	5
Content	Please indicate for each of the 5 statements which is closest to how you have been feeling over the past 2 weeks. Over the past 2 weeks… … I have felt cheerful and in good spirits …I have felt calm and relaxed …I have felt active and vigorous … I woke up feeling fresh and rested … my daily life has been filled with things that interest me Response categories: All of the time Most of the time More than half the time Less than half the time Some of the time At no time
*Diabetes Eating Problem Survey-Revised (DEPS-R)*	
Number of items	16
Content	Living with diabetes can sometimes be difficult, particularly regarding eating and diabetes management. Listed below are a variety of attitudes and behaviors regarding diabetes management. For each statement, tick the **one** answer that indicates how often this was true for you during the **past month** How often this was true for you during the past month… Losing weight is an important goal for me I skip meals and/or snacks Other people have told me that my eating is out of control When I overeat, I don’t take enough insulin to cover the food I eat more when I am alone than when I am with others I feel that it’s difficult to lose weight and control my diabetes at the same time I avoid checking my blood sugar when I feel like it is out of range I make myself vomit I try to keep me blood sugar high so that I will lose weight I try to eat to the point of spilling ketones in my urine I feel fat when I take all of my insulin Other people tell me to take better care of my diabetes After I overeat, I skip my next insulin dose I feel that my eating is out of control I alternate between eating very little and eating huge amounts I would rather be thin than to have good control of my diabetes Response categories: Never Rarely Sometimes Often Usually Always
*Questions on involvement and overall satisfaction*	
Number of items	6
Content	These questions are about the degree to which you have felt involved in your treatment (select only one answer for each statement) Patient’s experience of involvement: The health staff asked questions about my own experiences with my illness/condition I talked to the health staff about the questions or concerns I had The health staff encouraged me to ask questions or talk about my concerns I was involved when decisions were made about what was to take place I have had an appropriate number of talks with the health staff about how I can best handle my illness/condition All in all, I am satisfied with my last visit to the outpatient clinic Response categories: Not applicable To a slight degree To some degree To a great degree To a very high degree Don't know
*Additional comments area*	
Content	Write here if you have any comments on the questions or if you want to discuss something at the consultation

## Abbreviations

DEPS-R: Diabetes eating problem survey-revised
ID: Interpretive description
PROs: Patient-reported outcomes
WHO-5: World health organization-five well-being index
